# In Primiparous Women at Term, a Symphysial Fundal Height of 36 cm Is Linked to the Lowest Risk of Emergency Caesarean Section and Postpartum Haemorrhage

**DOI:** 10.1111/ajo.70113

**Published:** 2026-04-01

**Authors:** John Robert Salmon, Ashlee Jarrett

**Affiliations:** ^1^ University of New South Wales ‐ Albury Campus Wodonga Victoria Australia; ^2^ University of New South Wales Albury New South Wales Australia

**Keywords:** admission to special care nursery, emergency caesarean section, instrumental delivery, postpartum haemorrhage, symphysial fundal height

## Abstract

**Background:**

Guidelines for managing a small symphysial fundal height (SFH) measurement during pregnancy are well established, but guidelines identifying and managing a large SFH measurement at term are not.

**Aims:**

To determine the relationship between a large SFH at term and intra‐partum risk, and to determine if there is an SFH cut‐off that could predict the following adverse outcomes: emergency caesarean section (CS), instrumental delivery, postpartum haemorrhage (PPH) and admission of neonate to the special care nursery (SCN).

**Materials and Methods:**

A retrospective audit was performed on 775 deliveries at a regional Australian hospital. SFH measurement at term was the primary variable for investigation. The largest measurement taken within 14 days of delivery was accepted as the primary SFH measurement.

**Results:**

In primiparous women, an SFH of 36 cm was associated with the lowest incidence of emergency CS (8%) and 35 cm was associated with the lowest incidence of PPH (8%). Both risks trebled at an SFH of 40 cm (24%) and quintupled at 43 cm (42%). In multiparous women the emergency CS rate was not correlated with SFH, but the PPH rate was correlated.

**Conclusions:**

A SFH measurement at term is currently a neglected but useful measurement for triaging women early in labour. An SFH of 36 cm is most reassuring as the parturient is at low risk of an emergency CS or a PPH whereas a SFH of 40 cm or more indicates increased risk and intra‐partum care should be modified to reflect this increased risk.

## 1 Introduction and Historical Overview

The first documented use of symphysial fundal height (SFH) measurement was in 1906 by McDonald [1], as a means of determining the length of a pregnancy. Subsequently McDonald [2] described how she calculated the duration of pregnancy in lunar months by dividing the height of the uterus in centimetres by 3.5. She claimed this gave ‘the most exact means of estimation of the duration of pregnancy’. She also claimed that a cut‐off SFH is clinically useful. As she explained ‘it is still more useful for the determination of the size of the foetus, with a view to induction of labour for contracted pelvis or other cause. When the fundal measurement is at or near 35 cm, I never hesitate to induce labour when indicated, knowing that there is a foetus of the average weight of about 3300 g and capable of standing instrumental delivery and not liable to die from prematurity’.

The utility of SFH for estimating gestational age has not stood the test of time. A 2022 meta‐analysis concluded SFH is too inaccurate for estimating gestational age and should not be used for this purpose [3].

In the 1960s, SFH measurements found a new use, as a means of detecting poor foetal growth. Westin [4] developed the first SFH chart, based on 100 uncomplicated pregnancies and subsequently SFH curves, based on 50 large for dates (LFD) and 50 small for dates (SFD) infants. He then tested the chart on 428 women delivering between the 37th and 42nd week of pregnancy. The chart correctly identified 75% of all SFD infants and 65% of LFD infants. Thereafter SFH charts were developed worldwide for local populations, for example Taylor, Coulthard and Robinson [5] developed the first SFH chart for an Australian population.

Gardosi and Chang [6] introduced customised SFH growth charts adjusted for maternal height, weight, ethnicity and parity with the purpose of improving detection of the SFD foetus. The Perinatal Institute [7] developed this further with an online version called the ‘Gestation Related Optimal Weight’ GROW‐Chart which became the central focus of their ‘Growth Assessment Protocol (GAP) program’. Jayawardena and Sheehan [7] reviewed the introduction of the GAP program at a Melbourne tertiary referral centre and showed it increased the identification of SFD infants from 20% to 41% with no increase in the false positive rate.

Arguments citing the impreciseness of SFH measurements such as significant inter and intra‐observer variation persist [3]. This is not surprising when you consider variables such as maternal obesity, the foetal lie, Braxton Hicks contractions, the fullness of the woman's bladder, hydramnios, pelvic tumours and foetal movements all affect the measurement. Despite these concerns, medico‐legal pressures to support the continued use of SFH measurements in antenatal care remain. For example, Sinni et al. [8], in addressing the high medico‐legal liability associated with obstetric care, concluded that an SFH should be measured at every visit after 20 weeks gestation. They also noted that the rate of non‐compliance was low at 7.9%. Gibbons et al. [9] surveyed policies for monitoring of foetal growth in Australian and New Zealand Hospitals delivering 1000 births or more and determined that abdominal palpation and SFH measurement were the most used screening tools.

The National Institute for Health and Care Excellence (NICE) guidelines 2021 continue to recommend SFH measurement at each antenatal appointment after 24 weeks for women with a singleton pregnancy, unless the woman is having regular growth scans, and to plot the measurement onto a growth chart [10]. The current RANZCOG guidelines recommend the SFH be plotted on a customised antenatal growth chart and that early pregnancy risk selection is undertaken for foetal growth restriction with serial scanning recommended for women with specific risk factors [11].

Despite some reservations about the reliability of SFH measurements, SFH measurements continue to be widely accepted as having a useful role alongside ultrasound as a means of detecting poor foetal growth. However, large SFH measurements at term are mostly ignored. Our thesis is that a large SFH measurement at term also deserves attention, as it helps identify women at increased intra‐partum risk. Our retrospective study was designed to investigate the validity of this observation and to determine if we could identify a clinically useful SFH that could be used to establish a parturient's risks of adverse outcomes. We chose birth by emergency caesarean section (CS), instrumental delivery, PPH and admission to SCN as our adverse outcomes.

## 2 Materials and Methods

A retrospective audit was performed of 775 deliveries over a 7‐month period from 1 July 2014 to 31 January 2015 at Albury Wodonga Health (AWH)—Wodonga Campus. AWH is located on the New South Wales—Victorian state border. The hospital services a population of 105 000 and hosts 1700 births annually. Eligibility criteria included singleton pregnancy and delivery at 37 weeks 0 days or later.

A list comprising medical record numbers of all births in the specified time frame was used to search the computerised hospital database and to create the study database. SFH measurement at term was the primary factor for investigation. To ensure consistency, the largest measurement taken within 14 days of delivery was accepted as the primary SFH measurement. Measurements were sourced from electronic antenatal care notes, antenatal hand‐held records, emergency department triage and midwife care clinic records. Additional information collected included maternal age, height, weight, BMI, gestational weight gain (GWG) and parity, onset of labour (spontaneous or induced), need for augmentation, mode of delivery, blood loss, gestation at birth, neonatal weight, head circumference, admission to SCN, and SFH measurements within 14 days of delivery.

This study obtained ethical approval by the University of New South Wales human Research Ethics Committee (HREC) as well as the Albury Wodonga Human Research Ethics Committee (AWHREC) (LNR‐21‐12‐2014). Data was entered on Excel and the two graphs were created on Excel Version 16.77.1. Statistical analysis was performed using R Version 4.3.3.

The initial database included 998 cases. For the purposes of this study, we excluded the following cases:

1. All elective CSs (*n* = 165).
2. Maternal height of 152 cm or less (*n* = 29) as this cohort is known to be at high risk of disproportion. In our series 14/29 (48%) required a caesarean and 4/29 (14%) required an instrumental delivery.
3. All women who had no SFH recorded within 14 days of birth (*n* = 33).

Our final cohort of 775 cases was further separated into primiparous (*n* = 343) and multiparous (*n* = 432) groups. We then divided each cohort into 1 cm SFH groups and calculated the incidence of (1) emergency caesarean, (2) instrumental delivery, (3) postpartum haemorrhage (PPH) defined as blood loss of 500 mL or more and (4) admission to special care nursery (SCN).

Using Kruskal–Wallis sum test for statistical analysis, we showed that in the primiparous cohort, increasing SFH was a strongly correlated with increased incidence of emergency CS and increasing incidence of PPH, but no such correlation was evident with rate of instrumental delivery or rate of admission to SCN. In the multiparous group, increasing SFH was correlated with increasing incidence of PPH but no correlation was seen with the incidence of emergency CS, instrumental delivery or admission to SCN. We therefore excluded instrumental delivery and admission to SCN from the remaining analysis.

**GRAPH 1:**
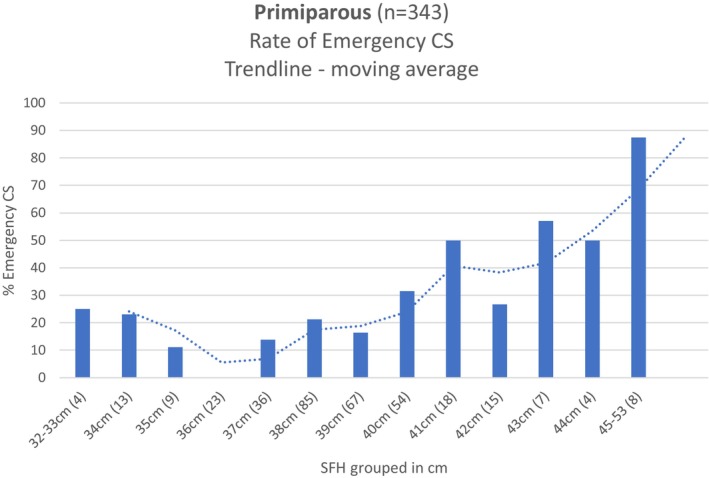
Primiparous (*n* = 343)—Incidence of emergency CS in different SFH groups and the moving average trendline.

**GRAPH 2:**
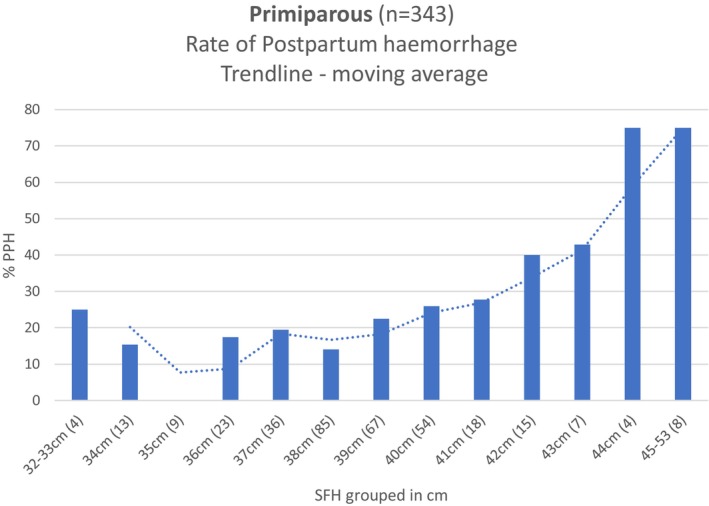
Primiparous (*n* = 343)—Incidence of Postpartum Haemorrhage in different SFH groups and the moving average trendline.

We proceeded as follows:

**Primiparous:** As discussed, we divided the primiparous cohort (*n* = 343) in 1 cm SFH groups and calculated the incidence of emergency CS and PPH for each SFH group. See Table 1 and Graphs 1 and 2.

**TABLE 1:** Incidence of adverse outcomes (emergency CS, PPH) in different SFH groups primiparous (*n* = 343) and multiparous (*n* = 432).

SFH	Primiparous (*n* = 343)			Multiparous (*n* = 432)		
	*n*	Emergency CS rate	PPH rate	*n*	Emergency CS rate	PPH rate
All SFH	343	82 (23.9%)	79 (23.0%)	432	27 (6.3%)	73 (16.9%)
32–33 cm	4	1 (25%)	1 (25%)	11	0	1 (9.1%)
34 cm	13	3 (23.1%)	2 (15.4%)	5	0	1 (20%)
35 cm	9	1 (11.1%)	0%	12	1 (8.3%)	0
36 cm	23	0	4 (17.4%)	28	1 (3.6%)	3 (10.7%)
37 cm	36	5 (13.9%)	7 (19.4%)	54	5 (9.3%)	7 (13%)
38 cm	85	18 (21.2%)	12 (14.1%)	96	4 (4.2%)	15 (15.6%)
39 cm	67	11 (16.4%)	15 (22.4%)	83	5 (6%)	12 (14.5%)
40 cm	54	17 (31.5%)	14 (25.9%)	66	3 (4.5%)	13 (19.7%)
41 cm	18	9 (50%)	5 (27.8%)	34	3 (8.8%)	11 (32.3%)
42 cm	15	4 (26.7%)	6 (40%)	20	1 (5%)	4 (20%)
43 cm	7	4 (57%)	3 (42.9%)	6	0	1 (16.7%)
44 cm	4	2 (50%)	3 (75%)	9	2 (22%)	3 (33%)
45–53 cm	8	7 (87.5%)	6 (75%)	8	2 (25%)	2 (25%)

A statistical relationship between SFH (continuous variable) and emergency caesarean (Yes/No) was observed in the primiparous cohort using the Kruskal–Wallis rank sum test. Kruskal–Wallis chi‐squared 22.364, *p* < 0.001 (SFH median 38 cm for vaginal birth, SFH median 40 cm for emergency CS). To present the data in a meaningful manner, and to compensate for the low numbers at either end of the SFH spectrum, we graphed the results and created a trendline which evens out fluctuations. We chose a Moving Average Trendline with the period option of 2 as this provided the best fit for the U‐shaped graph. This showed the lowest incidence of emergency caesarean (8%) occurs at a SFH of 36 cm, and this increases to 24% at 40 cm, and 43% at 43 cm.

Similarly, a statistical relationship between SFH (continuous variable) and PPH of 500 mL or greater (Yes/No) in the primiparous cohort was observed using Kruskal–Wallis Rank Sum test. Kruskal–Wallis chi‐squared 9.8007, *p* = 0.0017 (SFH median 38 cm for < 500 mL blood loss, SFH median 39 cm for > 500 mL blood loss). The moving Average Trendline shows the lowest incidence of PPH (8%) occurs at a SFH of 35 cm, and this increases to 25% at 40 cm and 42% at 43 cm.

**Multiparous**—Table 1: We repeated the same analysis on the multiparous cohort (*n* = 432). The relationship between SFH and emergency caesarean was not significant on Kruskal–Wallis sum test (Kruskal–Wallis chi‐squared 1.3504, *p* = 0.2451). Using a moving average trendline (not illustrated), the risk of an emergency caesarean is 4% at a SFH of 35 cm, remains 4% at SFH of 40 cm, and increases marginally to 7% at 43 cm. However, the relationship between SFH and PPH was significant, Kruskal–Wallis chi‐squared 7.9446, *p* = 0.0048 (SFH median is 38.6 cm for < 500 mL, 39.5 cm for > 500 mL). Using a moving average trendline (not illustrated), the incidence of PPH is 10% at a SFH of 35 cm, increases to 18% at 40 cm and remains 18% at 43 cm.

To determine which maternal and neonatal factors were significant contributors to the higher incidence of emergency CS and PPH, we analysed the primiparous group by performing the following: We used the primiparous mean SFH (38.7 cm) to divide the group into two cohorts, SFH ≤ 38 cm (*n* = 170) and SFH ≥ 39 cm (*n* = 173), see Table 2. This showed that a larger SFH was generally associated with a larger birth weight, a larger neonatal head circumference, a longer gestation, a greater pre‐pregnancy maternal weight, a greater BMI and a greater GWG. There was no association between a larger SFH and neonatal 1 min Apgar scores, maternal age or maternal height.

**TABLE 2:** Comparison of maternal and neonatal variables associated with a SFH ≤ 38 and ≥ 39 cm, primiparous (*n* = 343).

	SFH ≤ 38 cm (*n* = 170) 50%	SFH ≥ 39 cm (*n* = 173) 50%	Kruskal–Wallis rank sum test
Median neonatal			
Birth weight (g)	3206	3692	*p* < 0.001 (significant)
Head circumference (range 31–37 cm)	34	35	*p* < 0.001
Gestation at birth (weeks)	39.33	40.13	*p* < 0.001
Apgar at 1 min	8.49	8.27	Not significant
Median maternal			
Age (years)	26.93	27.01	Not significant
Height (cm)	166	166	Not significant
Weight pre‐pregnancy (kg)	65.9	73.3	*0* < 0.001
BMI	24	26	*p* < 0.001
Gestational weight gain (kg)	12.7	15.3	*0* < 0.001
Estimated blood loss (ml)	404	447	*p* < 0.05

## 3 Discussion

In our population of primiparous women, the moving average of ‘35 and 36 cm combined’ was associated with the lowest incidence of emergency CS (8%) and 35 cm with the lowest incidence of PPH (8%). This supports McDonald's observations 120 years ago. The risk of an emergency CS, 8% at 36 cm, doubles (16%) at 38 cm, trebles (24%) at 40 cm, quintuples (42%) at 43 cm. The risk for PPH is 8% at 35 cm, doubles (16%) at 38 cm, trebles (24%) at 40 cm, quintuples (42%) at 43 cm and increases nine times (75%) at 44–50 cm. We were reassured to see there was no correlation between SFH and Apgar scores or admission of neonate to SCN.

In the multiparous cohort, the emergency CS rate remains unchanged (6%) right up to 43 cm, but it trebled when the SFH exceeded 44 cm (18%). The multiparous PPH rate shows a gentler trend, that is, 10% at 35 cm, 14% at 38 cm, 17% at 40 cm, 18% at 43 cm, treble (29%) at 44–50 cm.^1^

Our study supports the supposition that the larger the SFH at the end of pregnancy, the greater the likelihood of birthing complications. Our data show that in primiparous women, a large SFH at term correlates with larger birth weight, larger neonatal head circumference, longer gestation, greater pre‐pregnancy maternal weight, greater maternal BMI and greater GWG. The importance of GWG is uncertain, but excess GWG may be a proxy for a less favourable maternal metabolism, which is known to adversely affect the strength of uterine contractions [12].

Failure to progress in labour is a common indication for an intra‐partum caesarean or instrumental delivery. Cephalopelvic disproportion (CPD) is sometimes to blame, and numerous strategies have been proposed to detect this before labour, such as antepartum ultrasound measurements to diagnose foetal macrosomia, radiographic imaging to identify pelvic contracture and various formulas to predict the arrest of labour [13, 14, 15]. None of these strategies have stood the test of time. Although a large SFH measurement at term cannot predict CPD, it is a quick, simple measure that successfully predicts an increased risk of needing an emergency CS.

Knowing the SFH measurement at term is useful information for allocating resources. By checking the most recently recorded SFH in the antenatal record, obstetric carers are offered a final opportunity to review a parturient's risk status early in labour. We propose in primiparous women that an SFH of ≤ 39 cm is reassuring and an SFH of ≥ 40 cm indicates the patient is at increased risk of a difficult birth.

In conclusion, SFH measurements continue to be useful in the provision of quality antenatal care. In addition to identifying SFD pregnancies, LFD SFH measurements at term are also useful. In the primipara, an SFH of 36 cm at term suggests the lowest risk of emergency caesarean and/or PPH, whereas an SFH of 40 cm or more is of concern, and therefore pre and intra‐partum care should be adjusted accordingly.

## Funding

The authors have nothing to report.

## Conflicts of Interest

The authors declare no conflicts of interest.

## Endnotes

^1^
The absence of a correlation in the multiparous cohort, between increasing SFH (up to SFH of 43 cm) and emergency CS, is likely explained by the higher elective CS rate (22% in multiparous, 6.9% in primiparous). Multiparous women at risk of a repeat difficult birth eg previous emergency CS, are more likely to be confined by an elective CS, therby diluting out high risk patients in the multiparous cohort.
